# Displaced Midshaft Clavicular Fractures among Patients with Clavicular Fracture Admitted to the Orthopaedic Department of a Tertiary Care Centre: A Descriptive Cross-sectional Study

**DOI:** 10.31729/jnma.8066

**Published:** 2023-03-31

**Authors:** Prabin Nepal, Netra Bahadur Karki, Kumar Shrestha, Umash Karki, Samir KC, Sailendra Kumar Duwal Shrestha

**Affiliations:** 1 Department of Orthopaedics, Nepal Armed Police Force Hospital, Balambu, Kathmandu, Nepal

**Keywords:** *clavicle*, *fracture*, *open fracture reduction*, *orthopedics*

## Abstract

**Introduction:**

Midshaft clavicular fractures are common among young adults. Open reduction and internal fixation of displaced midshaft clavicular fractures with plate and screws have been shown to decrease nonunion, symptomatic malunion, and residual shoulder disability compared with nonoperative treatment allowing early pain-free movement and early return to work. The aim of this study was to find out the prevalence of displaced midshaft clavicular fractures among patients with clavicular fractures admitted to the orthopaedics department of a tertiary care centre.

**Methods:**

A descriptive cross-sectional study was conducted in the Department of Orthopedics of a tertiary care centre from 31 January 2016 to 31 December 2019 after receiving ethical approval from the Institutional Review Committee (Reference number: 659/2021 P). Data were collected using hospital-based records from patients of the age group 18 to 50 years. Convenience sampling method was used. Point estimate and 95% confidence interval were calculated.

**Results:**

Among 120 patients, the prevalence of displaced midshaft clavicular fractures was 40 (33.33%) (24.90-41.76, 95% Confidence Interval). Among them 39 (90%) were males and 4 (10%) were females and the mean age of 31.45 years. The average Constant-Murley score were 95.68±5.59.

**Conclusions:**

The prevalence of displaced midshaft clavicular fractures among patients with clavicular fractures admitted to the Department of Orthopedics was lower than the other studies done in similar settings.

## INTRODUCTION

Midshaft clavicle fracture is one of the most common injuries around the shoulder girdle that occurs most frequently in individuals younger than 50 years. Displaced fractures have been treated both by conservative and surgical methods.^1,2^

Treatment of displaced clavicular fractures conservatively in an arm sling, clavicular brace or a figure of eight bandages can lead to a higher incidence of re-displacement, malunion, non-union, shortening and decreased shoulder strength.^3,4^ Thus, it has an impact on the short and long-term quality of life in active people because of the societal implications of work absenteeism or the need for modification of regular duties. Open reduction and internal fixation with pre-contoured locking plates enable an anatomical reduction, and stable fixation restoring the normal biomechanics of the shoulder girdle along with earlier mobilization.^5^

The aim of this study was to find out the prevalence of displaced midshaft clavicular fractures among patients with clavicular fractures admitted to the orthopaedics department of a tertiary care centre.

## METHODS

This descriptive cross-sectional study was conducted at the Department of Orthopedics, Nepal Armed Police Force Hospital, Kathmandu, Nepal from 31 January 2016 to 31 December 2019. Ethical clearance was taken from the Institutional Review Committee (Reference number: 659/2021 P). All patients of the 18 to 50 years age group were included in this study. Patients of age <18 and >50 years, with fractures treated with an IM device, compound fracture, floating shoulder, neurovascular injury, multiple fractures and pathological fractures were excluded from the study. Convenience sampling method was used. The sample size was calculated using the formula:


$$\begin{array}{ll} \text{n} & ={\text{Z}}^{2}\times \frac{\text{p}\times \text{q}}{{\text{e}}^{\text{2}}}\\ & =1.96^{2}\times \frac{0.654\times 0.346}{0.09^{2}}\\ & =108 \end{array}$$

Where

n = minimum required sample size
Z = 1.96 at 95% Confidence interval (CI)
p = prevalence of midshaft fracture, 65.40%^6^
q = 1-p
e = margin of error, 9%

The calculated sample size was 108. However, 120 patients were included in the study.

After obtaining informed consent, all patients were operated on under brachial plexus block/general anaesthesia. Patients were discharged after 5 days of uneventful postoperative stay in the hospital. Patients were followed up on the fourteenth postoperative day for suture removal. Physiotherapy was done progressively as per Ellis and Griffin's protocol.^7^ Functional outcome was assessed from recorded data using the Constant and Murley scoring system. The Constant-Murley score (CMS) is a 100-point scale to determine the functionality after the treatment of clavicle fractures.^8,9^

The collected data were entered into and analysed using Microsoft Excel version 16. Point estimate and 95% CI were calculated.

## RESULTS

Among 120 patients, the prevalence of displaced midshaft clavicular fractures was 40 (33.33%) (24.9041.76, 95% CI). The excellent outcome was observed in 33 (82.5%) of cases, good in 6 (15%) of cases and fair in 1 (2.5%) of cases in the one-year follow-up (Table 1). The mean time of union was 19 weeks ranging from 12 to 24 weeks. None of the cases had malunion or nonunion. Among them 1 (2.5%) presented with a broken implant at 3 months postoperative period without any obvious history of trauma. A total of 5 (12.5%) patients developed minor complications out of which three (7.5%) patients developed prominent hardware, one (2.5%) had sensory loss over the scar site and the other developed superficial infection which was treated with intravenous and oral antibiotics.

**Table 1:** Interpretation of Constant Scores (n= 40).

Category	Reference	3 months n (%)	6 months n (%)	12 months n (%)
Poor	<70	9 (22.50)	-	-
Fair	70-79	30 (75)	7 (17.50)	1 (2.50)
Good	80-89	1 (2.50)	26 (66)	6 (15)
Excellent	90-100	-	7 (17.50)	33 (82.50)

The average constant score at 3 months, 6 months and 1 year follow up was 71.18±10.90, 82.92±5.57 and 95.68±5.58 respectively. The mean age of the patients was 31.45±4.97 years, with a higher frequency of males 36 (90%) than females 4 (10%). The commonest mechanism of injury was fall from height, 20 (50%) and the commonest fracture type was Robinson's type 2B2, 22 (55%) (Table 2).

**Table 2:** Demographic profile of the study (n= 40).

Characteristics	n (%)
**Sex**	
Male	36 (90)
Female	4 (10)
**Mechanism of injury**	
Fall	20 (50)
RTA	19 (47.50)
Sports	1 (2.5)
**Side of injury**	
Left side	22 (55)
Right side	18 (45)
**Fracture type (Robinson)**	
Type 2B1	18 (45)
Type 2B2	22 (55)

## DISCUSSION

The prevalence of midshaft injury in our study was found to be 33.33% which is lower than the one of the studies in which the prevalence was 65.4%. Followed by this the prevalence was found to be higher in males (90%) in our study and the other similar study showed a prevalence of 70.6%, which is slightly lower than our finding.^6^ Among the different types of clavicle fractures, the middle third of the clavicular shaft fractures is the most common.^2,10,11^ Open reduction and internal fixation by locking plates allow proper fixation for bony union, allowing early mobilisation and better functional outcomes as shown by different studies.^10,12-14^

The most common mode of injury was fall injury (50%) followed by RTA (47%) and sports injury (3%) in our study. This was in contrast to the studies where road traffic accident,^15,16^ and sports injury was the most common cause of injury.^11^ This might be associated with more occurrences of fall injuries during training activities of paramilitary personnel in our study.

The prevalence of Robinson type 2B1 and type 2B2 was 45% and 55% respectively whereas, in one of the studies, the prevalence was 27.9% and 15.2% respectively.6 Our study showed excellent functional outcomes in 82.50% of cases and no cases of nonunion or malunion which was similar to the results shown by studies.^4,17-19^ In our study, the mean age of the patients was 31.45±4.97 years. This finding was similar to various similar studies suggesting the higher prevalence of clavicular fractures in young and active populations.^16,19,20^ The mean duration of union was 19 weeks which was similar to studies done in similar settings.^19,21-23^

In our study, the functional outcome score after open reduction and internal fixation at final follow-up was (95.67±5.58) according to the Constant-Murley scoring system. This finding was similar to the other studies conducted in similar settings in Nepal.^22-25^ Higher constant score was also shown in published studies (93.7).^26,11^

In our study, there was one major complication associated with implant failure at 3 months of the postoperative period. A similar complication in the same duration was observed in a study.^27^ Some of the likely factors responsible for the implant breakage were, the use of a plate in bridging mode, abnormal stress on the clavicle and highly motivated patients for early functional regain as mentioned in different studies.^27,28^ Patient was re-operated by using a longer locking plate and bone grafting. Regarding minor complications, one patient developed a superficial wound infection which was treated by intravenous and oral antibiotics. A similar complication was noted in studies done in similar settings.^11,25,29,30^ One patient in our study developed numbness over the scar site for up to 3 months which was resolved at the final follow-up. This finding was similar to a study where cutaneous hypoesthesia following plate fixation occurred which resolved gradually over a period of time.^31^ Hardware prominence and irritation were developed in three patients in our study owing to thin soft tissue envelope as noted in similar studies.^24,32^

The limitation to this study is that the finding can not be generalised to the larger population of the country and a study with larger sample size is recommended.

## CONCLUSIONS

The prevalence of displaced midshaft clavicular fractures among patients with clavicular fractures admitted to the orthopaedics department was lower than the other studies done in similar settings. An appropriate option for treatment modality should be chosen for a good result. A large multicentric prospective study is recommended for the further evaluation of the treatment outcome.

## References

[1] Stanley D, Trowbridge EA, Norris SH (1988). The mechanism of clavicular fracture. A clinical and biomechanical analysis.. The Journal of bone and joint surgery. British volume..

[2] Wiesel B, Nagda S, Mehta S, Churchill R (2018). Management of midshaft clavicle fractures in adults.. JAAOS-Journal of the American Academy of Orthopaedic Surgeons..

[3] Axelrod DE, Ekhtiari S, Bozzo A, Bhandari M, Johal H (2020). What is the best evidence for management of displaced midshaft clavicle fractures? A systematic review and network meta-analysis of 22 randomized controlled trials.. Clinical orthopaedics and related research..

[4] Naveen BM, Joshi GR, Harikrishnan B (2017). Management of mid-shaft clavicular fractures: comparison between non-operative treatment and plate fixation in 60 patients.. Strategies Trauma Limb Reconstr..

[5] Kingsly P, Sathish M, Ismail NDM (2019). Comparative analysis of functional outcome of anatomical precontoured locking plate versus reconstruction plate in the management of displaced midshaft clavicular fractures.. J Orthop Surg (Hong Kong)..

[6] Kihlstrom C, Moller M, Lonn K, Wolf O (2017). Clavicle fractures: epidemiology, classification and treatment of 2 422 fractures in the Swedish Fracture Register; an observational study.. BMC Musculoskelet Disord..

[7] John Ellis R, Schaper LA, Smith MG, Jeffrey Popham G, Nawab A, Salamon M (2020). Clavicle fracture ORIF protocol (Dr.. Sean Griffin) [Internet]..

[8] Constant CR, Murley AH (1987). A clinical method of functional assessment of the shoulder.. Clinical orthopaedics and related research..

[9] Locking FB, Osteosynthesis CP (2015). Indian Journal of Orthopaedics Surgery.. Indian Journal of Orthopaedics..

[10] Wang XH, Guo WJ, Li A, Cheng GJ, Lei T, Zhao YM (2015). Operative versus nonoperative treatment for displaced midshaft clavicle fractures: a meta-analysis based on current evidence.. Clinics..

[11] S Thyagarajan D, Day M, Dent C, Williams R, Evans R (2009). Treatment of mid-shaft clavicle fractures: A comparative study.. Int J Shoulder Surg..

[12] Xu CP, Li X, Cui Z, Diao XC, Yu B (2013). Should displaced midshaft clavicular fractures be treated surgically? A meta-analysis based on current evidence.. European Journal of Orthopaedic Surgery & Traumatology..

[13] Gheorghiu D, Sinopidis C, Brown DJ (2013). Treatment of acute clavicle fractures with an anatomical congruent clavicle plate.. Glob J Surg..

[14] Robertson C, Celestre P, Mahar A, Schwartz A (2009). Reconstruction plates for stabilization of mid-shaft clavicle fractures: differences between nonlocked and locked plates in two different positions.. Journal of Shoulder and Elbow Surgery..

[15] Postacchini F, Gumina S, De Santis P, Albo F (2002). Epidemiology of clavicle fractures.. J Shoulder Elbow Surg..

[16] Sitaula J, Regmi AP, Prajapati A, Thapa SB, Sharma BD, Shrestha SR (2018). Functional outcome of plating for displaced middle third clavicle fractures in adults.. Journal of Chitwan Medical College..

[17] Yan MZ, Yuen WS, Yeung SC, Wing-Yin CW, Wong SC, Si-Qi WW (2022). Operative management of midshaft clavicle fractures demonstrates better long-term outcomes: A systematic review and meta-analysis of randomised controlled trials.. PloS one.

[18] Lake N, Mombell KW, Bernstein E, O'Mary K, Scott J, Deafenbaugh B (2020). Improved functional outcomes following operative treatment of midshaft clavicle fractures in an active duty population.. Cureus.

[19] Prabhu Ethiraj D, Parvataneni Prathap D, Arun HS, Nagakumar JS (2016). Functional outcome in surgical management of midshaft clavicle fractures fixed with precontoured plate in adults.. International Journal of Orthopaedics..

[20] Bostman O, Manninen M, Pihlajamaki H (1997). Complications of plate fixation in fresh displaced midclavicular fractures.. Journal of Trauma and Acute Care Surgery..

[21] Cho CH, Song KS, Min BW, Bae KC, Lee KJ (2010). Operative treatment of clavicle midshaft fractures: comparison between reconstruction plate and reconstruction locking compression plate.. Clinics in orthopedic surgery..

[22] Dhoju D, Shrestha D, Parajuli NP, Shrestha R, Sharma V (2011). Operative fixation of displaced middle third clavicle (Edinburg Type 2) fracture with superior reconstruction plate osteosynthesis.. Kathmandu Univ Med J (KUMJ)..

[23] Shah G, Gurung G, Bista R, Paudel S, Sah DN (2020). Functional outcome of closed displaced clavicle fracture managed operatively with pre-contoured anatomical locking: A retrospective chart review.. Post-Graduate Medical Journal of NAMS..

[24] Jha GK, Timilsina P, Yadav D, Lamichhane S, Jha S (2018). Conservative Vs operative management of displaced midshaft clavicle fracture: A comparative study.. Biomedical Journal of Scientific & Technical Research..

[25] Onta PR, Sapkota K, Wahegaonkar K, Ranjeet N, Thapa P, Thapa UJ (2019). Treatment of midshaft clavicle fracture with anatomical contoured clavicular locking plate.. Asian Journal of Medical Sciences..

[26] Altamimi SA, McKee MD (2008). Nonoperative treatment compared with plate fixation of displaced midshaft clavicular fractures: Surgical technique.. JBJS..

[27] Claessen FM, Schol I, Ring D (2019). Unplanned operations and adverse events after surgery for diaphyseal fracture of the clavicle.. Archives of Bone and Joint Surgery..

[28] Batash R, Debi R, Grinberg D, Shema M, Elbaz A, Benedict Y (2019). Mechanical failure of plate breakage after open reduction and plate fixation of displaced midshaft clavicle fracture-a possible new risk factor: a case report.. Journal of Medical Case Reports..

[29] Wijdicks FJ, Van der Meijden OA, Millett PJ, Verleisdonk EJ, Houwert RM (2012). Systematic review of the complications of plate fixation of clavicle fractures.. Arch Orthop Trauma Surg..

[30] Attia ME, Zanfaly AI (2014). Plate fixation in midshaft fracture clavicle.. The Egyptian Orthopaedic Journal..

[31] Wang L, Ang M, Lee KT, Naidu G, Kwek EB (2014). Cutaneous hypoesthesia following plate fixation in clavicle fractures.. Indian Journal of Orthopaedics..

[32] Toogood P, Horst P, Samagh S, Feeley BT (2011). Clavicle fractures: a review of the literature and update on treatment.. The Physician and sportsmedicine..

